# Effective factors on voluntary childlessness and one-child tendency from couples’ perspective: Compulsory childlessness or child-avoidance? 

**DOI:** 10.22088/cjim.14.4.656

**Published:** 2023

**Authors:** Neda Ahmadzadeh Tori, Hamid Sharif-Nia, Fatemeh Ghaffari, Fereshteh Behmanesh, Abolghasem Pourreza

**Affiliations:** 1 Ph.D. in Health Education and Health Promotion, Social Determinants of Health (SDH) Research Center, Babol University of Medical Sciences, Babol, Iran.; 2 Traditional and Complementary Medicine Research Center, Addiction Institute, Mazandaran University of Medical Sciences, Sari, Iran; 3 Nursing Care Research Center, Health Research Institute, Babol University of Medical Sciences, Babol, Iran; 4 Social Determinants of Health Research Center, Health Research Institute, Babol University of Medical Sciences, Babol, Iran

**Keywords:** Voluntary childlessness, only child, Qualitative research.

## Abstract

**Background:**

Couples’ childless and one-child intention is one of the crucial challenges in all societies. Considering the aging of the world's population and the need to review birth control policies, it is necessary to take evidence-based measures. Therefore, the present study aimed to investigate the influencing factors on the tendency of couples to be childless and have only one child.

**Methods:**

The present study is the first part of a mixed (qualitative-quantitative) study. The study is qualitative with a conventional content analysis approach. The research population consists of all couples aged 15-49 in 2021 in Babol city, who were single or childless and had no intention of pregnancy in the future. Sampling is based on the purpose, and the number of samples is 40 couples. To collect data, face-to-face and semi-structured interviews were conducted with the participants.

**Results:**

The results of the analysis include 140 codes, 30 sub-categories, 9 categories

(Economic problems, uncertainty in the future security, threatened priorities, uncertainty about the continuation of life, Fear of becoming a parent, lack of support, diminishing religious beliefs, social role modeling and negative experiences) and two themes. These factors indicate the mandatory conditions for childlessness or one- child choice or voluntary child-free.

**Conclusion:**

The results revealed that voluntary childlessness and single-child depend on various aspects. Support of the government, parenting education and efforts to change the attitude of couples by the government can help to improve the health of the family and achieve the goals of population growth policies.

The study of the single child and childlessness phenomenon can be significant regarding both public and private perspectives. The change in the direction of demographic policies in recent years and the implementation of programs to encourage fertility show the importance of studying these phenomena from a public perspective. However, demographic policies in Iran and the world indicate that the role of private and individual aspects of this phenomenon, that is, moral acceptance and application of these policies in private and family life, is much broader and deeper than macro demographic policies (1). 

Today, people voluntarily decide not to have children, which is called voluntary childlessness. This means that people can have children but are childless and have no desire to have children, and they are reaching the final years of childbearing age (2). Although the Organization for Economic Co-operation and Development (OECD) states that the ideal average number of children is 2.2 for men and 3.2 for women, the tendency to have children has declined (3). In the past decades, societies have experienced shrinking families and increasing voluntary childlessness (4). 

The results of recent studies have shown that among some population groups, the tendency to have only one child and even not having children is visible (5). Statistics demonstrate the desire to have only one child and be childless is increasing in Iran, and most women want to postpone their first pregnancy or remain childless (6). 

The results of studies with a qualitative and quantitative approach show the reasons for voluntary childlessness with regard to several socio-economic and demographic characteristics in reducing childbearing, such as increasing women's education, educational and health policies, support from religious authorities, and reducing infant mortality, economic problems, increasing the distance between marriage and first pregnancy, raising the age of marriage, changing attitudes (7) researches with a lot of qualitative approaches (8) job, income, and educational level (8), religion, changing gender roles, new lifestyle and ideas (9) and also various cultural and social factors (9) such as the internet and social networks (10) have been involved.

The issue of childlessness and the decrease in the desire of people to have children is one of the aspects that has been affected by such conditions. Beck and Beck-Gersheim studied the fear and hope of people to have or not to have a child in people themselves and considered a change in individual values and ideas as the most important reasons for childlessness and a decrease in the desire to have children (11).

Today, in many countries, policymakers in the social and health fields are trying to encourage families to have children due to the negative population growth. However, couples do not want to have children for various reasons related to the political and economic situations and cultural conditions prevailing in different societies. This is while the tendency of couples to be childless and monogamous is associated with consequences such as increasing child dependence, feelings of loneliness, helplessness, dependence on parents, and more anxiety and worry in adulthood and the ever-increasing population decrease (12) and can lead to emotional damage in couples. Therefore, it is necessary to identify the influential factors in this phenomenon in different situations and societies in depth and use qualitative approaches to take evidence-based actions (13). According to Iran's new population policies based on increasing the population, the researcher decided to conduct a qualitative study to explain the causes of childlessness and voluntary single-childhood based on the views and experiences of couples. The results of this study can help family health policy makers, sociologists and health service providers at the regional and national level to identify the factors influencing the desire of couples to be childless and have only one child, so that in this way effective solutions can be found to maintain and designed and implemented to improve the health of society and the survival of the generation.

## Methods

The present study is the first part of an Exploratory Sequential Mixed Method study that was conducted with the permission of Tehran University of Medical Sciences (code: IR.TUMS.SPH.REC.1399.023) in 2021 in Babol city. The study is qualitative with a conventional content analysis approach. In the exploratory sequential mixed method, the researcher first begins with the qualitative phase of the research, examining the participants’ experiences (14).The inclusion criteria were to have at least fifth-grade education, healthy reproductive age of 18 to 49, the interval between marriage and the birth of the first child is more than five years or no intention of having children. The study's exclusion criteria were the samples' unwillingness to continue cooperation in each stage of the research. The research community involved childless and voluntarily single-child couples. In the data collection phase, after explaining the purpose of the study and obtaining written consent, semi-structured face-to-face interviews were conducted with 40 couples (table 1). 

The participants were selected using purposive sampling and the subjects were selected based on availability and convenience, according to the entry criteria and willingness of the subjects to cooperate in the research,and with maximum diversity in terms of age, sex, educational level, occupation, economic status, and social status. After the initial coordination, the interview with the participants was conducted in a quiet environment. A voice recorder was used to record the interviews. Some of the interview guide questions are included in the following:

"What made you decide not to have children anymore?" "In what situation would you have a child?" "In your opinion, what factors led to your voluntary choice to be childless?". What factors were involved in your choice to have only one child? he average duration of the interviews was 60 to 90 minutes, and the interviews continued until data saturation. To analyze the data, contractual content analysis was used according to Granheim and Lundman's method (15). Content analysis is one of the methods of analyzing qualitative studies by which the data are summarized, described, interpreted, and used to determine the dominant and main themes (14). Based on this, each interview was divided into meaningful units and coded (16). Then, the obtained codes were placed in categories and sub-categories (17). Data analysis was done at the same time as data collection. In this way, first, the interviews’ audio files were written in manuscripts. Then the researcher tried to find their evident and hidden elements by carefully studying the manuscripts and doing the initial coding. In this section, every written word and phrase was considered as a unit of analysis. Manuscripts and interpretive notes were simultaneously re-read, which helped identify initial connections between concepts extracted from participants' conversations. In the subsequent interviews, the codes of each interview were compared with each other and other codes of previous interviews to determine their similarities and differences. The notes and codes helped to shape the categories and sub-categories. With the progress of the interviews and the identification of the relationship between the classes, the main patterns and meanings within the interviews were identified. Categories were reviewed and compared with each other in several stages, and during data collection and analysis, some categories were merged as well as newer categories were created. Finally, during the analysis of the data obtained from the interviews, the research team reached an agreement about the data, sub-categories, categories, and themes.

**Scientific accuracy and validity of data: **In order to check the accuracy and correctness of the data, Guba and Lincoln's four criteria; Credibility, Dependability, Transferability, and Research Credibility of Confirmability were used (16). The researcher was in the field for 12 months. Different methods were used to collect data. Maximum variability was used in data collection. External observers confirmed the results. Participants reviewed the data. Also, all the stages of conducting the research, especially the stages of data analysis, were recorded in detail and in detail along the way.

**Ethical considerations:** The researcher started collecting data after visiting the research units, explaining the study’s objectives, and obtaining their written consent. Ethical issues regarding participant autonomy, confidentiality, and anonymity were addressed throughout the study. Also, a letter of introduction was received from the research Vice-Chancellor of the university to present to the research centers. Ethical approval was obtained from the Ethics Committee of Tehran University of Medical Sciences. This project results from a specialized doctoral thesis approved by the Tehran University of Medical Sciences with the number IR.TUMS.SPH.REC.1399.023.

**Table 1:** Individual characteristics of the couples participating in the study

**Code**	**Age(year)**	**Sex**	**Childless/single child**	**Education**	**Occupation**	**Marriage length (year)**
1	36	female	single child	diploma	housewife	14
2	36	male	single child	Associate Degree	Non-government job	14
3	32	female	single child	Middle School	housewife	17
4	34	male	single child	diploma	Non-government job	17
5	33	female	single child	Master	insurance employee	10
6	35	male	single child	Master	Hospital employee	10
7	34	female	single child	diploma	farmer	11
8	34	male	single child	diploma	Non-government job	11
9	32	female	single child	Bachelor	nurse	9
10	33	male	single child	Bachelor	teacher	9
11	33	female	single child	diploma	housewife	12
12	36	male	single child	diploma	Non-government job	12
13	34	female	single child	Bachelor	tailor	15
14	38	male	single child	Bachelor	security guard	15
15	37	female	single child	diploma	housewife	16
16	40	male	single child	Bachelor	Non-government job	16
17	32	female	single child	Bachelor	nurse	8
18	33	male	single child	Bachelor	Water and sewer employee	8
19	37	female	single child	Bachelor	lawyer	20
20	37	male	single child	Bachelor	engineer	20
21	39	female	single child	Middle School	farmer	17
22	41	male	single child	diploma	farmer	17
23	27	female	Childless	Bachelor	Labrator employee	5
24	30	male	Childless	Bachelor	Bank employee	5
25	27	female	Childless	Bachelor	housewife	6
26	27	male	Childless	Bachelor	School employee	6
27	32	female	Childless	Master	insurance employee	7
28	33	male	Childless	Master	insurance employee	7
29	27	female	Childless	PhD	employee	5
30	32	male	Childless	PhD	Academic staff	5
31	26	female	Childless	Bachelor	housewife	6
32	30	male	Childless	Master	lawyer	6
33	30	female	Childless	Master	nurse	7
34	32	male	Childless	Bachelor	engineer	7
35	31	female	Childless	Master	housewife	6
36	36	male	Childless	Master	engineer	6
37	40	female	Childless	Master	University accountant	9
38	42	male	Childless	diploma	Non-government job	9
39	39	female	Childless	Bachelor	Bank employee	12
40	38	male	Childless	Bachelor	Justice employee	12

## Results

The average age of the participants was 32.76±2.74 years. Other personal characteristics are presented in table 2. The results of the analysis include 140 codes, 30 sub-categories, 9 categories, including Family economic problems, Uncertainty in the future security of the child, threatened priorities, Uncertainty about the continuation of life, Fear of becoming a parent, Lack or weakness of support, Diminishing religious beliefs, Social modeling and Negative experiences of childbearing. Two themes were "individual limitations" and "social limitations.” Some of these factors, such as Lack of support, 

Family economic problems, and Uncertainty about the continuation of life, have led to the forced choice of childlessness and a single child. And some others, such as threatened priorities, weakening of beliefs and attitudes towards having children, etc., resulted in couples’ reluctance to have children (table 3). 

**Subject one: Individual limitations**: This theme includes five categories of "threatened priorities", " uncertainty about the continuation of life,” "negative experiences of having children,” "fear of becoming a parent,"and” diminishing religious beliefs”.

**Table 2:** Demographic characteristics of the studied couples

**Demographic characteristics**		**Number**
**Sex**	man	20
	woman	20
**Age**	25-29	6
	29-35	12
	Over 35	22
**Education**	Diploma	9
	Bachelor	21
	Master	8
	PhD	2
**Occupation**	Working	32
	House Keeping	6

**Table 3:** Sub-categories/categories and content of the extracted themes

**Sub-category**	**Category**	**Theme**
Maintaining appearance and beauty Convenience Social constraints caused by having children Fear of jeopardizing the academic position Fear of jeopardizing the job position	threatened priorities	Individual limitations
Lack of commitment of couples to each other Failure of one of the spouses to adhere to moral standards Marital conflicts and problems between couples Emotional Divorce	Uncertainty about the continuation of life	
A negative experience of childbirth A negative experience of pregnancy Unpleasant experiences of the child Limited sexual relations due to childbearing	Negative experiences of childbearing	
Anxieties of having children Birth phobia	Fear of becoming a parent	
Failure to pay attention to religious recommendations Lack of belief in the survival of the generation	Diminishing religious beliefs	
Inadequate financial condition Inability to meet child needs Career concerns	Family economic problems	Social limitations
Concern about the child's future Gender-related concerns Problems associated with substance abuse	Unsecured Future	
Lack of social support Lack of family support Lack of government support	Lack or weakness of support	
Being influenced by others Influence of media and virtual space	Social modeling for child avoidance	

**Threatened priorities: **Most participants believe that the fear of losing their current position is one of the influential factors in the tendency to remain childless and have only one child. The feeling of being threatened by losing their job, the possibility of continuing their education, and the desire to improve their career and education have made the participants choose the option of childlessness or having only one child. From these people’s perspectives, having a child is an obstacle to achieving their dreams and desires. According to these people, in today's society, the possibility of education for couples and their employment outside the home, as well as access to various entertainment facilities compared to the past, and the advancement of technology has caused them not to have enough time to have children. Some other participants stated they could not accept the responsibilities of keeping and raising a child. They often believe that all their life plans are abandoned under the burden of having children. Most of them admitted that one of the influential factors in voluntary childlessness and single child is the existence of suitable conditions for surrogacy and changing the rules of adoption in society. They also believed that access to such facilities had reduced their fear of infertility due to aging. Hence, they tend to focus on other priorities in their lives. Some of these participants believed that the limitation of independence and the feeling of backwardness is formed with the presence of a child in the family, and this is one of the reasons they do not want to have children.

"If the child comes, I have to be at home. I can't work anymore or travel or go out." (Code 1). "On the other hand, once my work assignment is clear, then I will think about having a child. I don't know if I will be officially employed or not. I am undecided. I don't want to have a child because of my circumstances." (Code 12).

I prefer to take care of myself. I want to travel and live for myself. I feel that having a child is a big obstacle for me in living my own life. There is an opportunity. If my wife gets older, we will use surrogacy. What is the need to put ourselves in the bondage of a child" (Code 40)? Participant women mentioned keeping fit and fear of obesity as factors influencing their reluctance to have children. "It's a nightmare for me to get fat after giving birth and not have this current body anymore" (Code 9). "I'm afraid of my belly getting bigger and my body getting out of shape" (code 6)."

**Uncertainty about the continuation of life: **An unfavorable family environment for having children is another compelling factor in the desire for childlessness and a single child. The couple's lack of commitment to each other, one of the couple's non-adherence to moral standards, unsolved marital conflicts, and the existence of problems between the couple have caused the couple to feel that having a child can force them into a forced life. Also, some participants stated that the previous pregnancy caused emotional distance from their spouses. Therefore, they tend to maintain their married life instead of having children. They see pregnancy again as a threat to their emotional life.

"I don't even know if I should say this or not. When I was nine months pregnant and breastfed my baby for 23 months, I felt my husband didn't like that I was pregnant. She distanced herself from me. This is while I needed his emotional attention and companionship "(13). From the point of view of some participants, the non-adherence of one of the couples to moral standards such as cheating, the propensity to promiscuity, and facing ethical and behavioral problems of one of the couples cannot be a good platform for having children. Some other speakers in their experiences mentioned the fear of change in the spouse’s personality and lack of satisfaction with married life as factors influencing the choice of having only one child and not having children.

"I am not sure about the choice of my husband. I don't know if he will be a 100% good father for my child or not. He is a person who changes every moment and has no stable personality. Because I do not hope to continue my life, if the child comes, I may have to burn and rebuild for the sake of the child. (Code 5). "I was thinking about all this, what if I fail in my marriage? I haven't reached stability yet. My position hasn't been solidified yet. I still haven't reached an understanding with my spouse. If I want to divorce later, the child will be sacrificed" (Code 2).

In the experiences of some other participants, the emotional divorce of couples cannot provide stable conditions in the family for having children. From their perspective, in a situation where love and affection, commitment and responsibility towards the other person's emotional feelings in the family are weak, having children is equivalent to sacrificing the future generation. The child cannot grow mentally, emotionally, and psychologically in this situation.

"Honestly, after the wedding, I regretted marrying my husband. I wanted to get a divorce. His morals were unbearable. But I had worked so hard to build a life together, and I was dying to leave my life. From the beginning, my husband did not have a good relationship with my family. He didn't even let me go to their house. That's why I wanted to get a divorce in the first nine months. We don't have feelings for each other. We live under the same roof. Having a child in this situation is just a destruction (code 3)".

"There is no husband who accompanies me and is by my side. Who listens to my words or fulfills my requests. To be only listener to my words. So that we could sit together and drink two cups of tea without any fight. To have peace between us, and understand the situation (Code 13)

**Negative experiences of having children: **Negative experiences from previous pregnancy and childbirth or unpleasant experiences from the child's growth and development have been involved in the choice of only one child by the participants of this study. According to the belief of a number of participants, during pregnancy and childbirth, because of the restrictions that are created in the sexual relations of couples, it creates an unpleasant experience of having children in couples. This choice was especially evident in women who experienced pregnancy at a young age. Negative and unpleasant experiences of having a child due to the mentioned problems, as well as facing the physical and psychological problems of the child after birth, have threatened the couple's motivation to have another child. In their experiences, some couples have cited problems such as lack of access to specialized health service providers during pregnancy as reasons for not wanting to have children again.

"When I was 9 months pregnant, plus 23 months of breastfeeding my baby, we didn't have any sexual relations. We didn't have any. It made me sad. My husband avoided me" (Code 27).

"Because of my financial problems, I had to give birth in a government hospital. All the students helped me give birth. The doctor did not visit me. I had a bad experience of giving birth in a government hospital. I don't want to be in such a situation again" (Code 1).

**Fear of becoming a parent: **Most participants stated that raising a child is accompanied by many difficulties. They often do not see the ability to be successful in their parental duties. The worries and fears of couples, because they need to be with their children full-time and also the fear of not being able to be the center of each other's attention as in the past despite the presence of children, were also mentioned as other reasons for their reluctance to have children. Some participants admitted that the fear of other people's interference in their child's upbringing, the fear that their child may not be responsible, or that their child may be affected by genetic diseases made them choose to have only one child or not to have children.

“What should we do if the child has genetic problems? This question is always in my mind. I have a cousin who has mental retardation or a cousin who has mental problems. I am very afraid that my child will have a genetic problem like them" (code) 3).

"I am afraid that my husband's attention will decrease. Because I saw this in my sister's life. This fear does not allow me to think about having children" (code 4).

**Diminishing religious beliefs** **: **In most participants’ view, having children is not one of the duties of couple’s duties. They believe that the generation’s survival should not be one of the consequences of marriage, and this traditional view should be changed. In other’s opinion, there is no room for religious advice on couples’ responsibilities to maintain survival of the generation.

"The survival of the generation is not important to me at all. I do not believe in religious beliefs relating to having children, and this is not important to me at all" (Code 5).

"I don't believe in religious advice and what the generation’s survival cost me. If I want to have a child at the age of 35 or not, and then I die at the age of 50, what does it matter if I want to think about these things" Code 7).

"Well, all people don’t need to have children. Should everyone have children? What did we do for our parents so that our children may do to us?" (Code 4).

**Theme two: social constraints: **This theme includes the four categories of family economic problems, uncertainty about the continuation of life, social modeling, and lack or weakness of support

**Family economic problems: **Economic problems and financial costs imposed on parents by having children are among the other essential factors the participants mentioned for not wanting children. From their point of view, having a child without solid financial support is a choice that will question their responsibilities as parents. Not having a stable job, not having housing and a steady income, or a small income due to the economic conditions prevailing in the society has caused couples to consider having children a risk. From their point of view, the necessity of having a child is the ability of parents to meet their and their child's basic needs even though they do not have this capability.

"I can ignore my needs, but if the child wants something, it can't be ignored. For example, you can say I won't eat this, I won't wear this, but you can't do it for the child. I'm unemployed; my current economic condition is unsuitable for having children" (Code 21).

"We can't support a child. My spouse and I have a monthly income of 4 million tomans, which I don't think is enough to cover the expenses of a child" (Code 6)

**Uncertainty in the future security of the child: **Concern for the child's future, concern for the child's upbringing and education, and lack of hope to provide for his welfare are among the reasons that have caused most couples not to have children. Economic instability in the family and unemployment of the head of the family has caused most of the participants in the study not to draw a bright future for themselves and their children.

"I am afraid of how to raise my child" (code 8).

"Education of the child is very important to me, for example, what work should he do? What school should he go to? What lessons should he study? What class should he go to?" (Code 2).

On the other hand, facing problems such as the high prevalence of delinquency and addiction among young people has caused couples to worry about their child being influenced by society.

"The child at school doesn't even have sexual safety. I see the news that you can't leave the child alone for a minute" (code 12).

"According to the current situation, the child's future is uncertain and stressful. I don't want to take risks" (code 12).

Some participants cited gender-related concerns as a factor in their reluctance to have children. They are afraid that the outcome of their pregnancy will be against their wishes due to the more difficult upbringing of boys than girls and the lack of social security, especially for girls. Some also believe that having a son or daughter causes them to live in worry caused by the social threats of the child.

"It is difficult to manage the child's future. If it is a girl, I am afraid of social insecurity. On the other hand, if it is a boy, it is difficult to be exposed to social threats such as smoking and other drugs" (Code 3).

"I am afraid of the dangers in the society. Especially the dangers that threaten the girl. It scares me to have children" (code 6).

**Social modeling: **Adopting a role model for having children from social media and its negative impact on the couple’s choice to have children was observed in some participants’ experiences. Transferring negative experiences of childbearing and negative advertisements for childbearing through social media are significant factors in changing couples' attitudes toward childbearing.

 "The movies and series that I watch often have a message about the harm that parents suffer because of having a child. The troubles, the loneliness of parents, and the many problems they have to endure for the sake of their children have demotivated me. 

I see that having a child is not only emotional and psychological support but also traumatic" (Code 4).

 "In media, I see that educated or even prosperous people have one child or they don't have one at all. I think to myself, why should I have a child?" (Code 21).

**Lack or weakness of support** **: **Most participants said that one of the reasons for their choice of childlessness and the single child was the lack of family or social support. Lack of access to kindergarten for working couples and lack of support from the employer are other reasons for not wanting to have children.

"My mother is not close to me. There is no one to help me in raising children. I think it is beyond my ability to do all the tasks of raising my children alone" (Code 4)."We are not insured. The employer does not allow me to come home a little earlier and take care of my child" (Code 8).

## Discussion

These categories show more general concepts called individual limitations and social limitations effective in the tendency of couples to be childless and have only one child. These factors indicate the mandatory conditions for having or abandoning children without compulsion.

Parsons et al. (2017) state that if one of the couples believes that a child will be an obstacle to achieving favorable conditions, he will have a negative attitude towards having children (17). Nevertheless, Kanani (2016) explains that many couples desire to have children but are in a situation where they inevitably choose the childless or have one child (1). From Miller et al.'s (1995) point of view, individual positive and negative motivations are essential for fertility. Positive motivations include the personal reasons of each person for wanting a child, like the joy of pregnancy, birth, and childhood, traditional views, parenting satisfaction, the feeling of need and survival, and the use of a child as a tool, and the negative motivations for fertility are the reasons for not wanting children such as fear of becoming a parent, parents' stress and child care challenges (18).

Social factors affecting pregnancy behavior have been stated in various studies, such as mother's employment, age of marriage, the number of children, access to contraceptives, living conditions, family income, type of acquaintance and marriage with a spouse, independence of women in the family, urban or being rural, the degree of industrialization, social development, ethnic and cultural beliefs and customs and even the society's view of the average number of family members (6, 9, 19-21). Xiawoei et al. (2004) state that the process of fertility transition is proportional to the developments that have occurred in various economic, social, and some traditional dimensions of the family, and it has caused changes in patterns related to marriage and, finally, behavior (22).

The results of the present study showed that one of the effective individual factors in the choice of childlessness and single child was threatened priorities. So the priorities of young couples are maintaining their physical appearance, having fun, living without the constraints of having children, getting rid of any parental responsibilities and duties, and having a high chance of separating from their spouses. Such prioritization is derived from a negative attitude towards having children, which requires very serious measures in this regard. In this context, Anderson et al. (2000) write that couples consider children as an obstacle for personal and marital progress, which affects the type of attitude towards childbearing. Some couples may believe that parenting is necessary to put aside personal wishes and desires and should dedicate their whole lives to their children (23). The results of the present study are consistent with the results of Soderberg et al. (2013) (24) and Manski and Mishar Ferdgarai (2003) (25). In today's society, most couples consider childbearing as an obstacle to their personal and social life, which is considered one of the pillars of the negative attitude toward childbearing. Today, women build their identity mostly through the social roles they play, and the roles of housekeeping and childbearing have lost their meaning and meaning (12). The results of Taqvai et al.'s research (2020) show that body management (body appearance and fitness management) is effective on women's attitudes towards fertility. In fact, the independent variable (whole body management) has explained 0.166 percent of the dependent variable (desire to have a child) (26).

The change in the social lifestyle has caused the pattern of childbearing to decrease, therefore, culture building through mass media by experts in the field of family health and sociologists can lead to a change in the views of couples towards having children. This category should be one of the priorities of policymakers, especially in Iran, where the policy of population increase is discussed. The results showed that another cause of childlessness and the single child was uncertainty about the continuation of life. Marital conflict shows the disharmony between couples, which has caused a break in their relationship and plays a significant role in their decision to accept childlessness and only one child (12).

The results of Behmanesh et al.'s study (2015) showed that the major conflicts between couples are related to the lack of trust in the spouse, the negative performance of one of the spouses in marital duties, and emotional divorce (27). Favorable conditions in the family increase the couple’s motivation to have children. Pre-marriage counseling, interventions such as resiliency training and how to deal with stress-causing factors in young couples, psychological counseling regarding the promotion of moral obligations of couples, and the promotion of a sense of responsibility towards each other may work in this field.

Negative experiences of having children were another effective factor in the tendency of couples to be childless and have only one child. The results of other studies show that having children is one of the factors in maintaining the stability and stability of marital relations (12, 27), which is not consistent with the results of the present study. Piranvand et al. (2017) state that women and even men fear pregnancy, childbirth, and the transition to the parenthood stage, which has caused most of their anxiety (28).

 Another effective factor in the couple’s tendency to be childless and have only one child been the fear of becoming a parent. The results of the study by Shapiro et al. (2014) also showed that the interference of having children with the mother's job and increasing the busyness of life are important factors in single-child (29). The results of Ahmadi et al.'s study also showed that couples' fear and worry, especially in women, are practical factors in choosing to have only one child or childlessness (10), which is consistent with the results of the present study. Receiving counseling by psychologists in pre-marital screenings can reduce couples' fear of becoming parents. Teaching couples how to raise children may decrease their fear of becoming parents.

Another category resulting from the data analysis in this study was the Diminishing religious beliefs, and attitudes towards childbearing. Religious tendencies and beliefs are effective factors in fertility (30). This means a direct and significant relationship existing between religious beliefs and increasing fertility. So firmer religious beliefs are associated with a greater desire to start having children at an early age and the number of children (30). The findings of Madiri's study (2017) show that the number of intended children in both sexes is affected by religiosity and gender attitudes, and religiosity is more effective than gender attitudes on the number of intentional children (31).

The results of the present study showed that family economic problems are among the other effective factors in the desire to have children. Job problems, especially from the point of view of the studied women, are considered important factors in the choice of childlessness and only child. This point of view may have been formed because some couples may believe that to have a child, they must first reach a certain level of career and financial progress, and they should not have children until such preparations are provided, and the child's well-being is ensured. The increase in women's desire to be in the labor market and work outside the home has decreased their tendency to have children and has impacted the decline in the rate of having children in society.

The women’s entrance into work and financial assistance to their spouses are effective in women's desire to have fewer children (12). In addition to the employment of women outside the home and their job problems despite the presence of children, the inability to provide for the needs of children has made people tend to have a single child and be childless. This means that the more families care about welfare, the desire to have children decreases (27, 32, 33).

Unguaranteed future for the child was one of the other compelling factors in the desire to be childless and have only one child. Worrying about the child's career and financial future, raising a child, feeling insecure in the community, concerned about the child's well-being (34), and worrying about increasing economic problems with the arrival of a child (35) are some of the factors that lead couples to be childless and have only one child. The results of the study by Kazemnejad et al. (2015) showed that economic problems are not related to the tendency to have children. This is because families with high income tend to have few children, which is inconsistent with the results of our study (33).

The difference in the results of the two studies may be related to the difference in the place, time, and economic status of the target group in the two studies. Extensive measures are needed in social security, reducing the burden of addiction and delinquency in society and youth employment. Another reason that has led to the choice of childlessness and only one child by the couples of the present study was the lack or weakness of support. The support received in social and family areas is vital in development. Support can be in the form of helping in child care, financial said companionship, and affection (36).

Social support in different dimensions is a key variable in childbearing in the research of Sadeghi and Saraei (2016). Hence, the percentage of the tendency to have children in the samples who had social support was much higher than in the people who did not have this support (37), it is necessary to formulate laws to force employers to allocate centers for women employees’ children to strengthen the motivation to have children in this group of couples. In addition, the government's financial support should turn from a slogan into a reality to increase the couple's desire to have a child. Encouraging families to take care of their children can also help. Adopting a role model to not having children is one of the other causes of couples' reluctance to have children. With the growing trend of communication and information technologies, virtual social media are a relatively new phenomenon that has caused changes in many aspects of human life. This change in the views and beliefs of couples about having children is also evident (12). Our findings are inconsistent with the results of the study by Saraei et al. (2013), which states that the internet has reduced the tendency to marriage and childbearing (32), and in compliance with the results of the study by Enayat et al. (2013) (38) that shows a significant relationship between the use of media (mobile network and virtual social networks) and the tendency to have children but the present study's results are inconsistent with the results of Ghorbani et al. (2016) (39).

This difference may be because the studied research population was only women and young people. The desire of families to have children is more dependent on cultural issues than economic variables. Therefore, the change of attitude in Iranian couples during the last few decades has caused many young couples to turn to single-child or childlessness (26). Therefore, due to the warning about the necessity of reforming Iran's demographic, it is necessary to take measures to change the attitude of couples towards having children at the national level. The domestic media knowingly or unknowingly beat the drum of one-child and individual-centeredness.

One child and childlessness in women and men are affected by the media, prioritizing oneself, fear of becoming a parent. Negative pressures, economic problems, fear of the future and uncertainty about the continuation of life due to problems with the spouse have caused that children are enough or they do not want children. As a result, cultivation, financial and welfare support of the government, parenting education, and comprehensive efforts to change the attitude of couples towards childbearing by policymakers in the field of family health and sociologists and the government can maintain and promote family health, achieve the goals of encouraging policies to increase the population and help prevent illegal abortions.

**Strengths: **The results of this research can help to identify the factors affecting the tendency towards childlessness and voluntary one-child in couples by the health policymakers of the society. Health policy makers' understanding of the current situation can help comprehensive planning at the regional and national levels to maintain and increase family health by changing couples’ attitudes towards childbearing.

**Limitations: **One of the limitations of the present study was the non-cooperation of men and even, in some cases, not allowing their wives to cooperate with the research team. The participants gave their time to the researchers according to their busyness and willingness to conduct the interview. Sometimes, due to their busy schedule, they did not tell all their material on the influential factors of fertility. In the present study, we omitted the selection criteria for infertile couples; childlessness should be by choice. Proving infertility in those who had never actively sought children was not easy. Hence, we had to rely on the participants' statements.
